# Genomic prediction models trained with historical records enable populating the German ex situ genebank bio-digital resource center of barley (*Hordeum* sp.) with information on resistances to soilborne barley mosaic viruses

**DOI:** 10.1007/s00122-021-03815-0

**Published:** 2021-03-25

**Authors:** Maria Y. Gonzalez, Yusheng Zhao, Yong Jiang, Nils Stein, Antje Habekuss, Jochen C. Reif, Albert W. Schulthess

**Affiliations:** 1 Leibniz Institute of Plant Genetics and Crop Plant Research (IPK), 06466 Gatersleben, Germany; 2 Department of Crop Sciences, Center for Integrated Breeding Research (CiBreed), Georg-August-University, Göttingen, Germany; 3 Julius Kühn Institute (Federal Research Centre for Cultivated Plants), Quedlinburg, Germany

## Abstract

**Key message:**

Genomic prediction with special weight of major genes is a valuable tool to populate bio-digital resource centers.

**Abstract:**

Phenotypic information of crop genetic resources is a prerequisite for an informed selection that aims to broaden the genetic base of the elite breeding pools. We investigated the potential of genomic prediction based on historical screening data of plant responses against the *Barley yellow mosaic viruses* for populating the bio-digital resource center of barley. Our study includes dense marker data for 3838 accessions of winter barley, and historical screening data of 1751 accessions for *Barley yellow mosaic virus* (BaYMV) and of 1771 accessions for *Barley mild mosaic virus* (BaMMV). Linear mixed models were fitted by considering combinations for the effects of genotypes, years, and locations. The best linear unbiased estimations displayed a broad spectrum of plant responses against BaYMV and BaMMV. Prediction abilities, computed as correlations between predictions and observed phenotypes of accessions, were low for the marker-assisted selection approach amounting to 0.42. In contrast, prediction abilities of genomic best linear unbiased predictions were high, with values of 0.62 for BaYMV and 0.64 for BaMMV. Prediction abilities of genomic prediction were improved by up to ~ 5% using W-BLUP, in which more weight is given to markers with significant major effects found by association mapping. Our results outline the utility of historical screening data and W-BLUP model to predict the performance of the non-phenotyped individuals in genebank collections. The presented strategy can be considered as part of the different approaches used in genebank genomics to valorize genetic resources for their usage in disease resistance breeding and research.

**Supplementary Information:**

The online version contains supplementary material available at 10.1007/s00122-021-03815-0.

## Introduction

Barley (*Hordeum vulgare* L.) is the fourth most important cereal crop worldwide. Sustainable barley production depends on the efficient use of valuable diversity in breeding. In this direction, genebanks have collected and preserved about half a million of barley accessions covering a large genetic diversity to improve crops (Sato et al. 2014). Nevertheless, the exploitation of this genetic diversity is limited, which is mainly due to the lack of breeding-relevant information on the accessions. Therefore, genebanks are encouraged to gather data on important agronomic and quality traits to enable the transformation of their collections into bio-digital resource centers (Mascher et al. 2019). Following the FAIR principles (Wilkinson et al. 2016), i.e., data should be findable, accessible, interoperable, and reusable, the Federal ex situ Genebank for Agricultural and Horticultural Plant Species hosted at the Leibniz Institute of Plant Genetics and Crop Plant Research (IPK Genebank) in Gatersleben, Germany, has made available comprehensive historical data, collected over seven decades for about ~ 13,000 accessions from each barley and wheat collections (González et al. 2018a; Gonzalez et al. 2018b; Philipp et al. 2019). The traits considered so far included thousand grain weight, flowering time, and plant height. Moreover, molecular passport data consisting of genotyping-by-sequencing (GBS) were generated for almost all barley accessions hosted at the IPK (Milner et al. 2019).

Information on the resistance of genetic resources to barley yellow mosaic virus disease would further increase the value of the bio-digital resource center. The disease is a serious threat to winter barley production in East Asia and Europe with yield loses of up to 50% (Plumb et al. 1986). Different strains of *Barley yellow mosaic virus* (BaYMV-1 and BaYMV-2) and *Barley mild mosaic virus* (BaMMV), transmitted by the vector *Polymyxa graminis,* cause the disease. Phenotyping of entire genebank collections for resistance to BaYMV and BaMMV in field trials is laborious and time-consuming. As an interesting alternative, allele mining has been successfully implemented in populations of ~ 2000 and 1090 barley accessions to identify novel donors of resistances for the cloned resistance genes *rym11* and *rym*4, respectively (Hofinger et al. 2011; Yang et al. 2014, 2017). Allele mining, however, is a limited approach as it searches for novel diversity exclusively at known functional or candidate genes loci and ignores valuable donors for novel unknown resistance genes. As a solution to this, Yu et al. (2016) suggested to combine in a first step phenotypic and genomic data of genebank material into a training population to calibrate a genome-wide best linear unbiased prediction model (GBLUP). Available molecular passport data can then be used in a second step to predict the phenotypes of entire genebank collections (Jiang et al. 2021).

Implementation of genome-wide prediction is straightforward for complex traits that follow the infinitesimal model (Barton et al. 2017; Meuwissen et al. 2001; VanRaden 2008; Whittaker et al. 2000). On the contrary, for traits which are controlled by a mix of a few major and many minor genes this is challenging. In such situations, it can be beneficial to include preexisting knowledge on known candidate genes to increase the accuracy of genome-wide prediction. This can be done either by defining specific weights of the relevant genes in the GBLUP model or by incorporating known genes as fixed effects (Bernardo 2014; Zhao et al. 2014).

The main goal of the present work was to investigate the potential of genomic prediction as a tool to add information to the bio-digital resource center of barley genetic resources hosted at the IPK Genebank using historical and already published non-orthogonal data of plant responses to barley yellow mosaic viruses (Milner et al. 2019). The specific objectives were to (1) investigate the potential of different genome-wide prediction models exploiting different levels of phenotypic data from historic records, (2) compare genome-wide prediction and marker-assisted selection, and (3) optimize the number of major associations included into a genomic prediction approach that can bridge the gap between genomic and marker-assisted selection.

## Materials and methods

### Phenotypic and genomic data

**Fig. 1:**
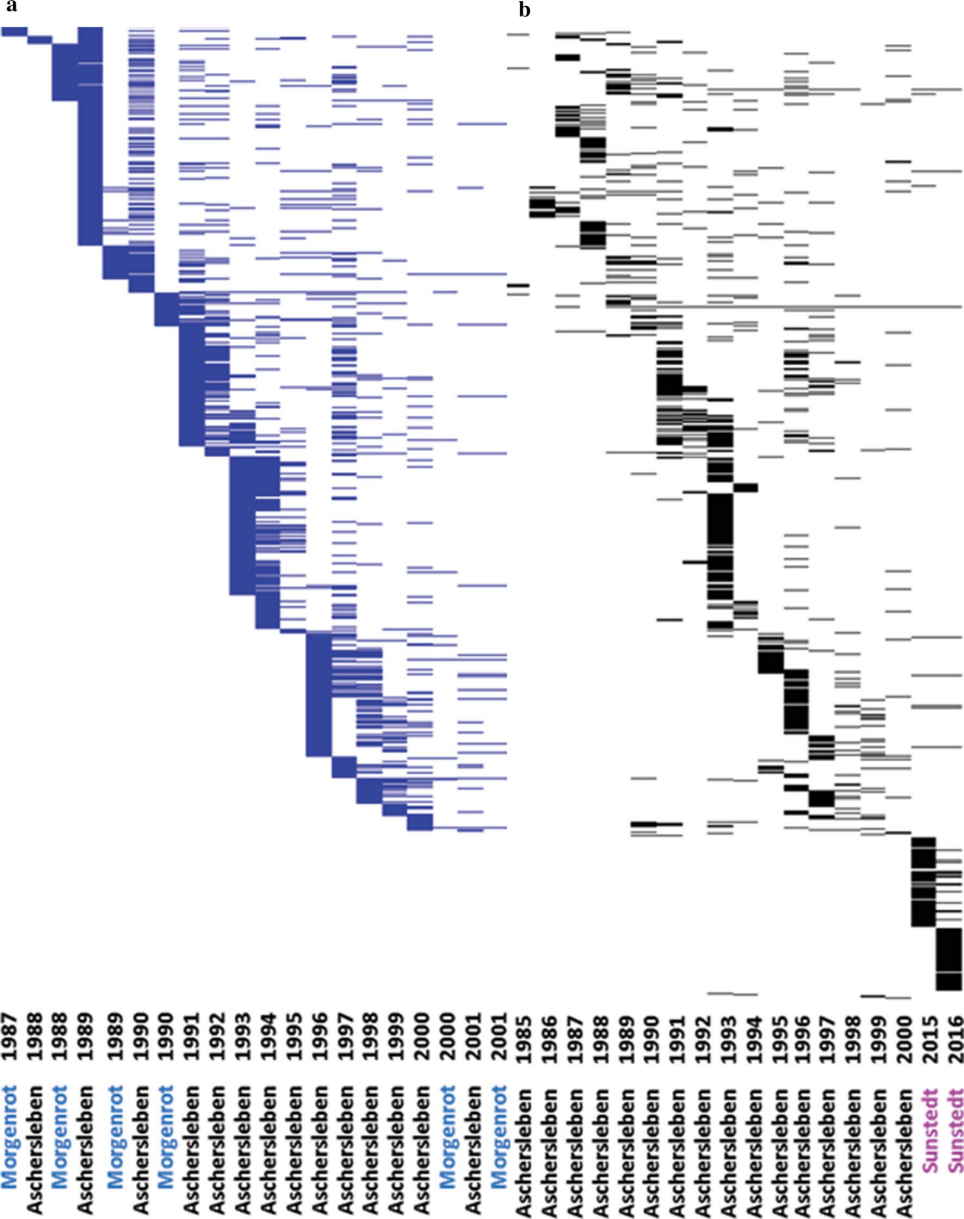
Layout of historical records of susceptibilities to barley yellow mosaic viruses: **a** BaYMV (blue) and **b** BaMMV (black) of 2083 winter barley accessions, which were phenotyped in Aschersleben, Morgenrot and Sunstedt during the period 1985–2016. The * y*-axis corresponds to the accessions, sorted by the first year in that a particular accession was tested (color figure online)

Our study is based on winter barley accessions (*Hordeum vulgare*) belonging to the ex situ collection of the IPK Genebank. In an attempt of mining new sources of resistance against key barley pathogens in Germany, the Federal Research Centre for Cultivated Plants (Julius Kühn Institute, JKI) and its predecessor organizations received each year a different set of accessions from the IPK Genebank for disease resistance screening. Over the years, the accumulated data formed a historical phenotypic data set. In more detail, we made use here of 15 and 18 years of historical screening data accumulated during the 1985–2016 period for susceptibility to the barley mosaic viruses: BaYMV and BaMMV, respectively. The data for BaYMV and BaMMV susceptibility were published and used in a previous study to show the potential use of genome-wide association analysis in genebank collections (Milner et al. 2019). Across years, BaYMV susceptibility was assessed in artificially inoculated field trials in Aschersleben (Saxony-Anhalt) and using natural infections in Morgenrot (Saxony-Anhalt). BaMMV susceptibility was evaluated in Aschersleben and Sunstedt (Lower Saxony) relying on mechanical (Habekuß et al. 2008) and natural infections, respectively. In all field trials, the experimental unit corresponded to an observation plot of 1 m^2^ size and the sowing date was in September. Mosaic symptoms of accessions were visually rated using a 1–9 scale, where 1 means complete absence of symptoms and 9 denotes completely susceptible. Even though mosaic symptoms were recorded for some accessions more than once during the season, we only had access to minimum, average, and maximum scoring values for each accession. In this regard, we based our analyses only on the maximum scored value, because these records maximize the discrimination power between resistant and susceptible accessions. In addition, no form of blocking was observed in data sets; thus, we assumed that accessions were tested in trials using a completely randomized experimental design without replications. The absence of blocking within field trials allowed us to subtract the data points pertaining these 2083 winter accessions without the risk of disrupting important structure features of the data. The filtered phenotypic data used in our study included 4166 and 2601 records for 1751 and 1771 accessions on infection scorings for BaYMV and BaMMV susceptibility, respectively. The data are unbalanced across years, locations, and traits (Fig. 1) with a number of accessions per environment (combination year-location) ranging from 5 to 468.

In a recent study (Milner et al. 2019), 20,458 barley accessions of the IPK Genebank were characterized by GBS. Briefly, genomic DNA was digested with *PstI* and *MspI* (New England Biolabs) (Wendler et al. 2014), while sequencing was performed on the Illumina HiSeq 2500 using multiplexes of 180 barcoded samples. After read trimming, reads were mapped to the reference sequence of the barley cultivar Morex (Mascher et al. 2017). Later, SNP loci with more than 90% missing data, more than 10% heterozygous calls or whose number of heterozygous calls exceeded the number of homozygous calls for either allele, were discarded. Using this marker matrix, missing genotype calls were imputed using FILLIN (Swarts et al. 2014). After imputation, 306,049 SNPs with more than 90% present calls and minor allele frequencies (MAF) > 1% were kept in the matrix. The remaining up to 10% missing values per SNP in this matrix were filled in using the respective MAF values. SNP matrices data have been deposited at https://doi.org/10.5447/IPK/2018/9 (Mascher 2018).

Out of the 20,458 barley accessions, 2083 winter-type accessions were also present in the historical data set. According to the passport data (Milner et al. 2019), accessions with phenotypic records and genetic profiles correspond to 76% six-rowed, 13% two-rowed, 4% intermedium, 6% non-classified, and 0.3% deficiens. The accessions were collected in 44 geographic places worldwide. Collection hot spots were located in the Soviet Union (15%), Turkey (14%), Korea (11%), Germany (9%), the USA (6%), and Japan (6%).

### Phenotypic analyses

Based on a mixed-model (Henderson 1975) plus outlier correction strategy recently implemented to deal with the lack of orthogonality of historical phenotypic records (González et al. 2018a; Philipp et al. 2018), the best linear unbiased estimations (BLUEs) were computed for each trait. Four mixed models were fitted by considering combinations for the effects of genotypes, years and locations.

1 $${\varvec{y}} = 1_{r} \mu + {\mathbf{Zg}} + {\mathbf{Eu}} + \user2{e,}$$

2 $$\user2{ y} = 1_{r} \mu + {\mathbf{Zg}} + \user2{e,}$$

3 $$\varvec{y} = 1_{r} \mu + {\mathbf{Zg}} + {\mathbf{Eu}} + {\text{ }}{\mathbf{Wl}} + {\text{ }}\varvec{D\delta } + \varvec{e},$$

4 $${\varvec{y}} = 1_{r} \mu + {\mathbf{Zg}} + {\mathbf{Xv}} + {\varvec{e}},$$

where ${\varvec{y}}$ is the vector of phenotypic values of genotypes, $1_{{\varvec{r}}}$ denotes an r-dimensional vector of 1′s, and r is the number of records within ${\varvec{y}}$; $\mu$ is the common intercept term; ${\varvec{g}}$ indicates the vector of genotypic effects; ${\varvec{u}}$ corresponds to the vector of year effects; ${\varvec{l}}$ is the vector of locations effects; ${\varvec {\delta}}$ indicates the interaction between year and location; ${\varvec{v}}$ denotes the vector of environment effects; and ${\varvec{e}}$ is the vector of residuals. The design matrices allocating the elements of ${\varvec{g}}$**,** ${\varvec{u}}$, ${\varvec{l}}$, ${\varvec{\delta}},$ and ${\varvec {v}}$ to the ones of ${\varvec{y}}$ are ***Z***, ${\varvec{E}}$, ${\varvec{W}}$, ${\varvec{D}},$ and ${\varvec{X}}$, respectively. To obtain the BLUEs of genotypes, we assumed $\mu$ and ${\varvec{g}}$ as fixed parameters while the other parameters are random and normally distributed in the way: ${\varvec{u}} \sim N\left( {{\mathbf{0}},{\varvec{I}}\sigma_{U}^{2} } \right)$, ${\varvec{l}} \sim N\left( {0,{\varvec{I}}\sigma_{L}^{2} } \right)$, ${\varvec{\delta}} \sim N\left( {0,{\varvec{I}}\sigma_{\delta }^{2} } \right)$, ${\varvec{v}} \sim N\left( {0,{\varvec{I}}\sigma_{V}^{2} } \right)$ and ${\mathbf{e}}{ } \sim N\left( {0,{\mathbf{I}}\sigma_{R}^{2} } \right)$. Firstly, estimations across years were based on Eq. (1) for Aschersleben and Morgenrot, and Eq. (2) for Sunstedt. Afterward, Eqs. (3) and (4) were fitted on the raw data of BaYMV and BaMMV susceptibility for outlier detection, which was based on rescaled median absolute deviation and Bonferroni–Holm test (Bernal-Vasquez et al. 2016). This last approach flagged 21 and 157 data points as outliers for BaYMV and BaMMV susceptibility, respectively. By eliminating these data points, 32 accessions were completely removed for BaMMV susceptibility. Later, Eqs. (3) and (4) were refitted on depurated data sets to compute the BLUEs across years and locations for BaYMV and BaMMV susceptibility, respectively.

### Genetic diversity and linkage disequilibrium analyses

Pairwise Rogers’ distances (Rogers 1972) were calculated based on filtered SNP data as follows:

5 $$\frac{1}{p}\mathop \sum \limits_{s = 1}^{p} \sqrt {\frac{1}{2}\mathop \sum \limits_{t = 1}^{{n_{s} }} \left( {r_{{{\text{st}}}} - q_{st} } \right)^{2} } ,$$

where $r_{{{\text{st}}}}$ and $q_{{{\text{st}}}}$ are the allele frequencies of the $t$-${\text{th}}$ allele at the *s*-${\text{th}}$ locus in the two accessions under consideration, $n_{s}$ denotes the total number of alleles at the *s-*th locus, with $n_{s}$ = 2 in the case of SNPs. The distance consists on the average difference for the allelic frequency across all loci among two accessions, standardized with the factor $\sqrt {\frac{1}{2}}$ to restrict the values from 0 to 1. Extreme values 0 and 1 reflect full similarity and full divergence among accessions, respectively. Considering two homozygous lines P1 and P2, their F1, and O as a resulting inbred offspring derived from F1 cross, the Rogers’ distances fulfill two genetical properties as follows: First, the distance of F1 with either P1 or P2 is half of the distance between P1 and P2. Second, the distance of O with P1 or P2 is equal to the distance among P1 and P2 (Melchinger et al. 1991). In particular, the calculation of Rogers’ distance can be simplified in the case of SNP markers as follows: Let ${\varvec{M}} = \left( {m_{{{\text{is}}}} } \right)$ be the $n \times p$ matrix of SNPs ($1 \le i \le n, 1 \le s \le p$) coded as 0, 1, 2 which is the number of reference alleles. The Rogers’ distance matrix is an $n \times n$ matrix whose (*i*, *j*)-entry is given by $\frac{1}{2p}\mathop \sum \nolimits_{s = 1}^{p} \left| {m_{{{\text{is}}}} - m_{{{\text{js}}}} } \right|$.

Principal coordinate (PCo) analysis (Gower 1966) was applied on each of the Rogers’ distances matrices, and afterward, the first to fourth PCos were plotted against each other in order to portray the potential population structure due to geographic origins and row-type status of accessions. Pairwise linkage disequilibrium (LD) was calculated as the squared Pearson correlation (*r*^2^) between markers (Hill and Robertson 1968).

### Genome-wide association scans and prediction

Genome-wide association analysis (GWAS) was performed using the following linear mixed model (Yu et al. 2006):

6 $$\varvec{y} = {\mathbf{1}}_{\varvec{n}} \mu + {\text{ }}\varvec{X\beta } + \varvec{m}a + \varvec{g} + \varvec{e},$$

where ${\varvec{y}}$ is an $n \times 1$ vector having the phenotypic records of $n$ accessions, $1_{{\varvec{n}}}$ symbolizes an $n \times 1$ vector of 1 s, $\mu$ is a scalar and corresponds to the total population mean, ${\varvec{\beta}}$ denotes a $4 \times 1$ vector containing effects for different row types previously described (there were five different row types, resulting in four degrees of freedom for the row-type factor), whereas ${\varvec{X}}$ is an $n \times 4$ design matrix containing logical variables that assign elements of ${\varvec{\beta}}$ to ${\varvec{y}}$, ${\varvec{m}}$ and $a$ indicate the marker coding and the additive effect of the SNP being tested, respectively, ${\varvec{g}}$ is an $n \times 1$ vector containing the genetic background effect of accessions, while ${\varvec{e}}$ represents the $n \times 1$ vector of residual variation. For each SNP, reference (Morex) and alternative alleles in homozygous state were coded within $m$ as 1 and − 1, respectively, whereas the heterozygous state was coded as 0. The markers whose MAF exceeded the 1% threshold were considered for GWAS. In this regard, for GWAS without cross-validations and based on BLUEs across locations and years, BaYMV accounted for 1751 accessions and 253,838 markers, and BaYMV included 1739 accessions and 256,423 markers. In Eq. (6), $\mu$, ${\varvec{\beta}}$, and $a$ were assumed as fixed, while ${\varvec{g}}$ and ${\varvec{e}}$ were considered as random in the way ${\varvec{g}} \sim N\left( {0,{\varvec{G}}\sigma_{g}^{2} } \right)$ and ${\mathbf{e}}{ } \sim N\left( {0,{\mathbf{I}}\sigma_{e}^{2} } \right)$, respectively, where $0$ is a null vector of size $n$, ${\varvec{G}}$ denotes an $n \times n$ genomic estimated relationship matrix computed according to first method in VanRaden (2008), ${\mathbf{I}}$ is an $n \times n$ identity matrix, whereas $\sigma_{g}^{2}$ and $\sigma_{e}^{2}$ correspond to the genetic and error variance components, respectively. At each SNP position, the significance of a Wald statistic in the context of mixed models (Henderson 1975) was used to test whether the corresponding *a* effect was significantly different from 0. Genome-wide multiple testing was assessed by adjusting $P$-values using the method of Benjamini and Hochberg (1995), with the genome-wide false discovery rate (FDR) set at 5%. SNPs with significant associations were afterward all together included in a linear regression model to compute proportions of trait variance explained by them (*R*^2^).

Genome-wide prediction was performed using GBLUP (VanRaden 2008) and the weighted genome-wide best linear unbiased prediction (W-BLUP) (Zhao et al. 2014). Subsetting marker data according to the different levels of available phenotypic data introduced random fixation at some marker loci. These monomorphic markers were removed in order to perform GBLUP and W-BLUP. For instance, when genomic prediction accounted for BLUEs computed across locations and years, the data set for BaYMV accommodated 1751 accessions and 305,739 markers, and the data set for BaMMV included 1739 accessions and 305,788 markers. The mixed-model underlying GBLUP is obtained by dropping $\varvec{X\beta }$ and ${\text{ma}}$ terms from Eq. (6):

7 $${\varvec{y}} = 1_{{\varvec{n}}} \mu + {\varvec{g}} + {\varvec{e}}.$$

In the same manner as in Eq. (6), the genomic estimated relationship matrix for the random term ${\varvec{g}}$ was computed according to first method in VanRaden (2008). GBLUP is then extended to W-BLUP by adding information on the $p$ most significant markers that were found as associated with traits during genome-wide association scans:

8 $${\varvec{y}} = 1_{{\varvec{n}}} \mu + {\varvec{g}} + {\varvec{F}}_{{\varvec{G}}} {\varvec{g}}_{{\varvec{f}}} + {\varvec{e}},$$

where ${\varvec{g}}_{{\varvec{f}}}$ and ${\varvec{F}}_{{\varvec{G}}}$ stand for the $p \times 1$ vector of additive effects and the $n \times p$ design matrix of associated markers, respectively. Similar to GWAS, SNPs within ${\varvec{F}}_{{\varvec{G}}}$ were coded as − 1, 0, and 1, for the reference allele in homozygous state, heterozygous state, and alternative allele in homozygous state, correspondingly. In addition, genomic heritability was computed including all the accessions for the analysis as follows: $\frac{{\sigma_{g}^{2} }}{{\sigma_{g}^{2} + \sigma_{e}^{2} }}$, where $\sigma_{g}^{2}$ and $\sigma_{e}^{2}$ were estimated from Eq. (7). A resampling study using 100 random samples containing 80% of the accessions was performed to estimate the sampling variance of the genomic heritability. Finally, the genetic correlation between plant responses against BaYMV and BaMMV was estimated according to Schulthess et al. (2017) using a multiple-trait model. Linear mixed-model equations for genome-wide prediction were solved using the R package BGLR (Pérez and de los Campos 2014), whereas those of phenotypic, genome-wide association analyses and genetic correlation were computed using the mixed-model package ASReml-R (Butler et al. 2009).

### Cross-validated genomic predictions

Genomic heritability at the different levels of the phenotypic data as well as a general whole-genome association scan for each trait using BLUEs across locations and years for all accessions with phenotypic records were the only analyses performed without cross-validations. A cross-validation strategy was implemented under the frame of genome-wide prediction. In this context and for each of the two studied traits, a random sample containing 80% of the total number of accessions and the remaining 20% of them formed the training and test set, respectively. Accessions within the training set presented both, genomic and phenotypic data, while those within the test set conserved their genomic profiles but their phenotypic records were masked. Random sampling was repeated, producing 100 different combinations of test and training sets. In the case of GBLUP, the ${\varvec{G}}$ matrix was calculated for the whole population and subsequently used to fit Eq. (7) in each of the 100 runs. Three data levels were considered for the genomic prediction implementation as follows: (i) BLUEs across years and locations were used to perform genomic prediction with both, GBLUP and W-BLUP approaches, (ii) BLUEs across years, and (iii) data of years nested within locations were used only for GBLUP approach. W-BLUP can only be implemented after detecting markers with significant associations using GWAS. Therefore, whole-genome association scans were firstly run in each of the 100 generated training sets. In a second step, the first $p$ most significant markers of each run were used for W-BLUP as shown in Eq. (8) while using the same ${\varvec{G}}$ matrix calculated for GBLUP. For this, W-BLUP prediction ability was assessed based on six significant thresholds defined according to FDR-corrected *P*-values in the way: (i) the first, (ii) first 5, (iii) first 10, and (iv) first 20 SNPs having the lowest *P*-values for associations during GWAS, as well as all SNPs whose associations were significant at (v) $P$-value < 0.05 and (vi) $P$-value < 0.1. For each set of *p* markers, the effective number of independent associations (Gao et al. 2008) portrayed by them was computed as the number of principal components (PCs) needed to explain 95% of variation when principal component analysis is applied on the different size-*p* LD matrices. Afterward, we derived a relative non-redundant information content measurement by dividing the effective number of independent associations by its corresponding *p* and expressed this content as a ratio between 0 and 1. In addition, for each set of $p$ markers detected in each run, their MAFs as well as *R*^2^ values of GWAS were computed without sampling using complete data sets for each trait. Predictions were computed as $1_{n} \hat{\mu } + \hat{g}_{{{\text{GBLUP}}}}$ or $1_{n} \hat{\mu } + \hat{g}_{{{\text{W}} - {\text{BLUP}}}} + F_{G} \hat{g}_{f}$ for GBLUP and W-BLUP, respectively. For MAS, marker effects for each cross-validation run were estimated using linear regression on the training set data. These estimates were multiplied in the test set with the respective genomic profiles, while the sum of products across markers provided the corresponding prediction for each genotype. For GBLUP, W-BLUP, and MAS, prediction abilities were calculated as the Pearson correlation between predictions and observed phenotypes of accessions within each test set. During cross-validations, models were fitted using different levels of phenotypic data. GBLUP was implemented using either the BLUEs calculated across years and locations, the BLUEs computed for each location across years or records for years nested within locations. Nevertheless, due to the high computational burden of GWAS during cross-validations, W-BLUP was only implemented using BLUEs calculated across years and locations. The cross-validation strategy for MAS was implemented using BLUEs across years and locations for the six scenarios. Furthermore, we compared the performance for the non-phenotyped accessions based on MAS and W-BLUP accommodating an optimized number of main associations. All computational methods of this work were performed within R environment (R Core Team 2019) using R version 3.4.3.

## Results

### Historical data on susceptibility to barley yellow mosaic viruses showed a large phenotypic variation

The distributions of BLUEs over years and environments illustrated the broad spectrum of plant responses against BaYMV and BaMMV for the barley accessions studied (Table 1). Most accessions showed moderate susceptibility against BaYMV and BaMMV, which was reflected by mean infection scorings of 3.7 and 4.8. Genomic heritabilities of 0.47 for BaYMV and of 0.63 for BaMMV susceptibility evidenced the moderate contribution of the genetic component to variation of susceptibilities. Virus infection was also influenced by the locations, and this effect was more pronounced for BaMMV than for BaYMV susceptibility (Fig. S1). Specifically, accessions evaluated for BaYMV susceptibility in Morgenrot scored on average 0.8 points less on the evaluation scale than those tested in Aschersleben. Accessions assessed for BaMMV susceptibility in Aschersleben scored 2.3 points less on the evaluation score than those evaluated in Sunstedt. The moderate but positive correlation (*r* = 0.52, $P$-value < 0.001) between the BLUEs of the accessions for BaYMV and BaMMV susceptibility and the genetic correlation 0.81 suggest that the genetic mechanisms underlying both types of susceptibilities are partially shared.

**Table 1:** Number of accessions of winter barley, number of phenotypic records (total and average per accession), mean ± standard deviation, range, and coefficient of variation (CV) of the best linear unbiased estimations (BLUEs), and genomic heritability for BaYMV and BaMMV susceptibilities tested at three different German locations between 1985 and 2016

Trait		No. of accessions	Phenotypic records	Records per accession	Mean ± SD	Range	CV (%)	Genomic heritability	
								Mean	Sampling variance
BaYMV	Total	1751	4145	2.4	3.7 ± 2.5	− 0.7–11.5	67	0.48	0.0005
	Aschersleben	1740	3786	2.2	4.2 ± 2.5	− 0.7–11.7	60	0.42	0.0006
	Morgenrot	362	380	1.0	3.4 ± 3.0	0.99–9.0	88	0.41	0.0023
BaMMV	Total	1739	2444	1.4	4.8 ± 3.4	0.99–9.0	70	0.63	0.0007
	Aschersleben	1451	2202	1.5	4.4 ± 3.2	0.4–9.4	73	0.69	0.0007
	Sunstedt	344	399	1.2	6.7 ± 3.6	1.0–9.0	54	0.39	0.0037

### Inspecting BaYMV and BaMMV susceptibility and their relation to geographic origin and row type

**Fig. 2:**
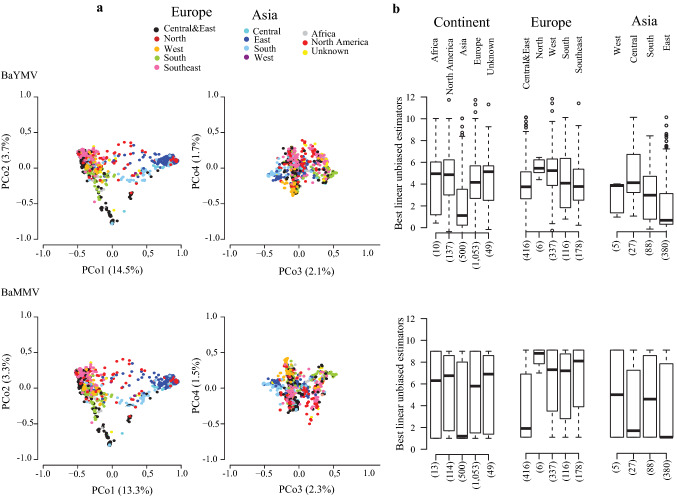
Genetic and phenotypic diversity as a function of geographic origins, presented for 2083 accessions evaluated for susceptibilities to mosaic viruses BaYMV (upper half) and BaMMV (lower half) in the period 1985–2016 at up to 2 locations. **a** Biplots taking into account the first four principal coordinates (PCo) from a PCo analysis performed on the Rogers’ distance matrix among accessions. The different colors represent the varied geographic origins according to the passport data of accessions. **b** Distributions of the best linear unbiased estimations (BLUEs) of accessions according to their geographic origins. The numbers in brackets refer to the total number of accessions in each geographic origin (color figure online)

The first PCo explained 14.5% and 13.3% of the total molecular variation of accessions tested for BaYMV and BaMMV susceptibility, respectively, and separated European from Asian accessions (Fig. 2a). A large amount of accessions from Asia, especially from East Asia, displayed low susceptibility to both BaYMV and BaMMV (Fig. 2b). Although highly variable, most European accessions showed weak to moderate susceptibilities to BaYMV. Despite that BaMMV infection also varied greatly among European accessions, a large proportion of the accessions from Central and East Europe showed low susceptibility, while most accessions with other European origins showed moderate to high susceptibility to BaMMV. PCo2 explained 3.7% and 3.3% of the whole molecular variation of accessions with BaYMV and BaMMV susceptibility records, respectively. This component tends to separate the East Asian accessions from the other Asian accessions. PCo3 explained 2.1% and 2.3% of the total molecular variation of accessions with BaYMV and BaMMV susceptibility records, respectively, while PCo4 accounted for 1.7% and 1.5% of molecular variation of accessions, correspondingly. PCo3 and PCo4 allowed a clear separation between six- and two-rowed accessions (Fig. S2). In general, high phenotypic variation including the complete range of plant responses to BaYMV and BaMMV was observed within most row-type groups. According to a Welch T-test ($P$-value < 0.01), six-rowed accessions presented significantly lower scores for BaMMV susceptibility than those having two rows.

### Assessing prediction ability using GBLUP

**Fig. 3:**
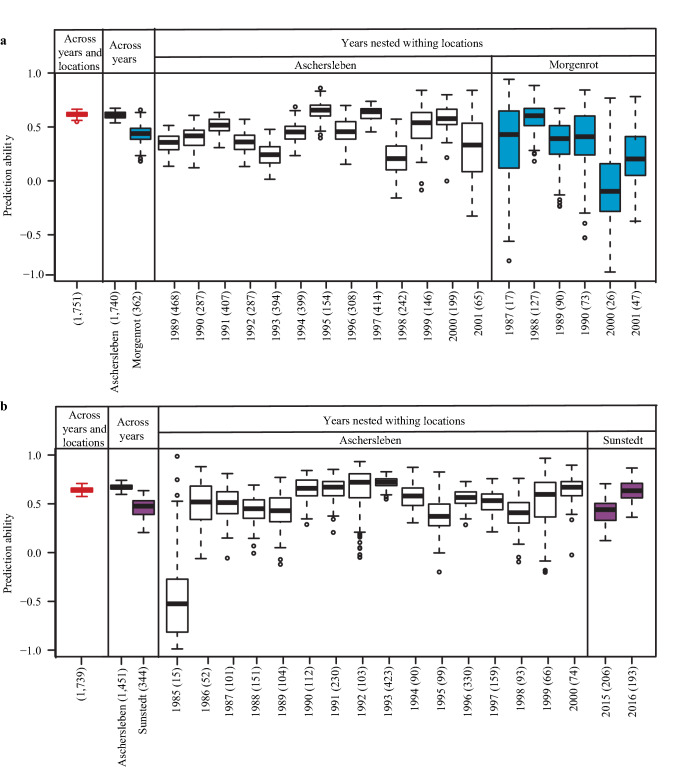
Cross-validated prediction abilities of genome-wide predictions using GBLUP for **a** BaYMV and **b** BaMMV susceptibility estimated based on three data levels (i) across locations and years, (ii) across years, and (iii) years nested within locations. The numbers in brackets refer to the total number of accessions in each data set

Because of the pronounced effects of locations on BaYMV and BaMMV susceptibility (Fig. S1), we have estimated the prediction ability of GBLUP not only for the total data set, but also within locations across years, as well as for each year–times–location combination separately. The prediction abilities were high for the total data set amounting to 0.62 for BaYMV and 0.64 for BaMMV susceptibility (Fig. 3). Separate consideration of the data for each location resulted in similar (Aschersleben) or slightly lower prediction abilities (Morgenrot, Sunstedt). This clearly suggests that a combined analysis across locations is not upwards biased due to stratification effects. The prediction abilities for data of years nested within the locations were highly variable, with the average for BaYMV susceptibility ranging from 0.21 to 0.65, and for BaMMV susceptibility from − 0.47 to 0.72. This can be explained by the sometimes low population sizes of individual location–times–year combination with a minimum of 15 genotypes.

### Genome-wide scan revealed markers significantly associated with BaYMV and BaMMV susceptibility

GWAS was performed for the entire data set and revealed 52 significant ($P$-value < 0.05) marker–trait associations for BaYMV and 64 for BaMMV susceptibility (Fig. S3). Significant associations were observed on 5 chromosomes with a hot spot on chromosome 3H containing a large number of marker–trait associations: 49 for BaYMV and 43 for BaMMV susceptibility. On chromosome 4H, we observed a second peak for BaMMV susceptibility with 16 marker–trait associations. The markers associated with chromosome 4H showed on average a higher LD than the markers associated with chromosome 3H (Fig. S4). In addition, markers associated with BaMMV susceptibility were limited to a 3.2 Mb region on chromosome 3H and a 0.37 Mb region on chromosome 4H. In contrast, markers associated with BaYMV susceptibility were located on a larger region covering 121.1 Mb on chromosome 3H (Fig. S4a). Further significant associations for BaMMV susceptibility were found on chromosomes 1H, 5H, and 6H (Fig. S3). Additionally, 3 SNPs on chromosome 6H were associated with BaYMV susceptibility. Each marker accounted for up to 7.5% of BaYMV and 8.8% of BaMMV susceptibility variation, while all markers together explained 32% for BaYMV and 25% of the variation for BaMMV susceptibility (Fig. S3–S4). Since Asian accessions were not only genetically distant to the rest of the accessions (mostly from European origins, Fig. 2a) but also less susceptible to both BaYMV and BaMMV in general (Fig. 2b), we investigated the shifts in significance by separately fitting a linear model for single-maker associations within the Asian and the rest of the characterized accessions (Fig. S5). It was observed that 92% and 65% of the markers associated with BaYMV and BaMMV, respectively, have lower $P$-values, i.e., are more significant, in the Non-Asian group compared to the Asian fraction. For those markers being more significant within the Asian pool, we observed a concomitant shift in the allele frequencies between Asian and Non-Asian groups (Fig. S6). Nevertheless, differences larger than 0.2 were only observed for 6% of markers associated with BaYMV and 3% of those associated with BaMMV. These results suggest that the observed statistical power for significant associations detected by GWAS in the total population depended on both Asian and Non-Asian groups, but relied mostly on the increased diversity and population size of the Non-Asian fraction.

### Assessing predictabilities of yellow mosaic virus using MAS

**Fig. 4:**
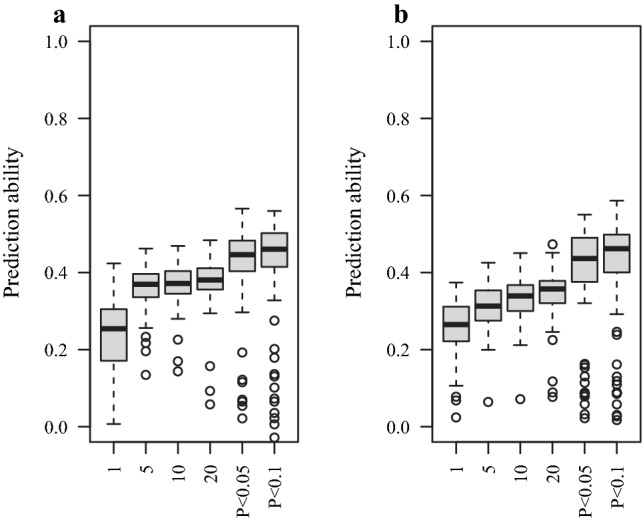
Cross-validated prediction abilities for **a** BaYMV and **b** BaMMV susceptibilities considering six different significant thresholds for associated markers: (i) the first, (ii) first 5, (iii) first 10, and (iv) first 20 most significant SNPs, as well as SNPs whose associations were significant at (v) $P$-value < 0.05 and (vi) $P$-value < 0.1

The prediction abilities for MAS computed in a linear regression model for six different significance thresholds revealed an inferior performance for MAS respecting GBLUP. On average, prediction abilities ranged from 0.24 to 0.38 for BaYMV and from 0.26 to 0.34 for BaMMV for thresholds including the first, (ii) first 5, (iii) first 10, and (iv) first 20 most significant SNPs from GWAS scans. The most relaxed thresholds of $P$-value < 0.05 and $P$-value < 0.1 showed the highest prediction abilities: 0.42 for BaYMV and 0.40 for BaMMV (Fig. 4). However, the highest prediction abilities for MAS were 32% and 38% lower for BaYMV and BaMMV, respectively, compared to those in GBLUP (Fig. 3). These results stimulated us to combine MAS and genomic prediction by assessing W-BLUP prediction abilities.

In cross-validations, average *R*^2^ values of markers approached zero by using more liberal significant thresholds (Fig. S7). Nevertheless, the highest average *R*^2^ values observed with the most conservative threshold, i.e., only considering the most significant marker, were never higher than 1.6% and 1.4% for BaYMV and BaMMV, respectively. These very low average *R*^2^ values observed in general were a consequence of the high number of spurious associations detected during cross-validations at any used significant threshold. For both BaYMV and BaMMV, average MAF values increased slightly with more liberal significant thresholds, while the average of the detection rate increased first progressively by relaxing the significance thresholds, peaking at the 20 most significant markers, but dropped afterward using the two most liberal thresholds of $P$-value < 0.05 and 0.1. We checked the correlation between MAF, *R*^2^, and detection rate in cross-validations (Fig. S8). While the detection rate was not correlated with MAF, low positive correlations were observed between MAF and *R*^2^ (*r* = 0.12* for BaYMV and BaMMV), and among detection rate and *R*^2^ (*r* = 0.35*** for BaYMV and *r* = 0.29*** for BaMMV). Accordingly, the most stable association for BaYMV was observed at marker 3:690,855,738, mapping on chromosome 3H, having a MAF of 0.14, a detection rate of 90.7%, and an *R*^2^ value of 7.5%. In addition, marker 3:691,483,734 mapped 0.62 Mb away from 3:690,855,738 and carried the second most stable association for BaYMV and the most stable one for BaMMV. This marker has a MAF of 0.21 and presented detection rates of 93.8% and 85% as well as *R*^2^ values of 8.8% and 6.4% for BaMMV and BaYMV, respectively.

### Assessing prediction abilities of yellow mosaic virus susceptibility using W-BLUP

**Fig. 5:**
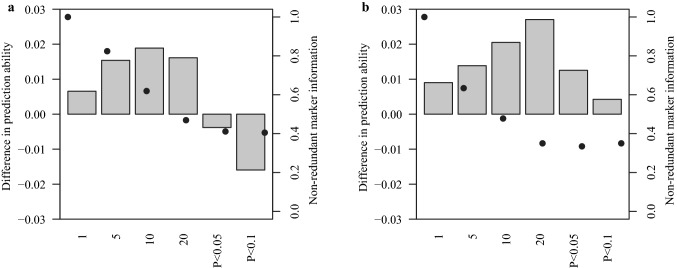
Average differences in cross-validated prediction abilities between GBLUP and W-BLUP for **a** BaYMV and **b** BaMMV susceptibilities considering six different significant thresholds for associated markers: (i) the first, (ii) first 5, (iii) first 10, and (iv) first 20 most significant SNPs, as well as SNPs whose associations were significant at (v) $P$-value < 0.05 and (vi) $P$-value < 0.1. Black dots indicate the average percentage of the total information provided by associated markers which are non-redundant (color figure online)

We contrasted the prediction ability of GBLUP with that of W-BLUP, which gives special weight to important diagnostic markers (Fig. 5). The W-BLUP model was evaluated considering six different significance thresholds for the selection of diagnostic markers. Within the range of the first twenty most significant markers, the prediction ability of W-BLUP was progressively increased for both traits compared to GBLUP by relaxing the significant threshold. The prediction ability of W-BLUP peaked at 10 markers for BaYMV susceptibility, resulting in a 0.02 increase (~ 3.0%) in prediction ability compared to GBLUP. In case of BaMMV susceptibility, this peak was observed at 20 markers, resulting in a 0.03 increase (~ 5.0%) in prediction ability over GBLUP. As previously mentioned, these peaks showed correspondingly the highest average detection rates for BaYMV and BaMMV susceptibility (Fig. S7). Furthermore, using the standard significant thresholds of $P$-value < 0.05 and $P$-value < 0.1 decreased the advantages of the prediction ability of W-BLUP over GBLUP for both traits. In this latter context, the more liberal the threshold, the greater the decrease in the prediction ability. The maximum decrease in prediction ability using standard significant thresholds was observed for BaYMV susceptibility, for which W-BLUP achieved even lower prediction abilities as compared to GBLUP. Furthermore, we observed that the ratio between the non-redundant genetic information and the total information provided by markers decreased until a plateau was reached when the thresholds became more liberal and more markers were fitted as fixed effect in W-BLUP. As a result, reaching this plateau was also almost simultaneous with the peak in prediction ability achieved by W-BLUP (Fig. 5).

### Breeding values estimated based on genomic prediction and marker-assisted selection for major loci

**Fig. 6:**
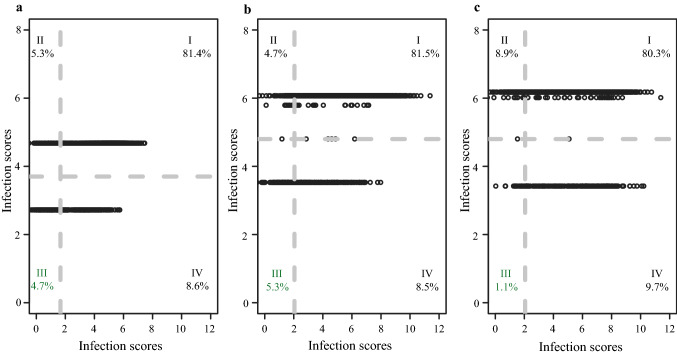
Comparison among genome-wide prediction using W-BLUP and marker-assisted selection (MAS) for non-phenotyped winter barley accessions maintained at the IPK genebank. W-BLUP based on first 10 SNPs for BaYMV, and first 20 SNPs for BaMMV having the lowest *P*-values for associations during GWAS. MAS stand for **a** the highest associated marker for BaYMV susceptibility and for the most associated markers at chromosomes 3H **b** and 4H **c** for BaMMV susceptibility. Each diagram was divided into I-IV quadrants, each quadrant included the respective percentage of accessions. Culling levels defined by vertical lines separated the 10% less susceptible genotypes detected by genomic prediction. Culling levels delineated by horizontal lines stand for the population mean, and separated the susceptible accessions (upper) from the less susceptible ones (lower) according to allelic effects in MAS

We compared the usefulness of MAS with that of genome-wide prediction from a breeder’s viewpoint. For this, we considered the most significant marker of chromosome 3H for both traits and on chromosome 4H for BaMMV and performed predictions using MAS and W-BLUP (Fig. 6). Predicted values were then plotted against each other to compare the selection accuracy of both methods. In first step, culling levels represented by vertical lines separated the 10% less susceptible genotypes detected by genomic prediction. Culling levels delineated by horizontal lines stand for the population mean and separated the susceptible accessions (upper) from the less susceptible ones (lower) according to allelic effects in MAS. Accordingly, culling levels formed four different quadrants (I to IV) that can be described as follows:

Quadrant (I) represents susceptible genotypes for both genomic prediction and MAS. It includes 81.4% of accessions for BaYMV, and 81.5% and 80.3% of accessions for BaMMV. Quadrant (II) stands for susceptible accessions according to MAS but classified in the top 10% as less susceptible according to genomic prediction. This quadrant comprehends 5.3% of accessions for BaYMV, and 4.7% and 8.9% of accessions for BaMMV. Quadrant (III) represents accessions that would be selected by both MAS and genomic selection, including 4.7% of the accessions for BaYMV, and 5.3% and 1.1% of accessions for BaMMV. Quadrant (IV) includes accessions that would be selected by MAS but fall outside of the selection criteria for genomic prediction. This involved 8.6% of accessions for BaYMV, and 8.5% and 9.7% of accessions for BaMMV.

The agronomical value of crop varieties comes from the combination of several traits into the same single cultivar. In order to have enough variation to breed for multiple traits using methods such as tandem selection or independent culling levels, plant breeders tend to relax their selection criteria for single traits (Bernardo 2010). It is very unlikely that a single accession from those selected as parents for pre-breeding already carries all desired trait values, but pre-breeders may also like to relax their selection criteria in order to avoid severe future bottlenecks in their programs. Particularly for BaYMV and BaMMV, this would mean to admit individuals with slightly to middle susceptibility values within the highly resistant selected fraction. The continuous nature of genomic predictions for BaYMV and BaMMV allows to shift culling levels in a flexible manner, while MAS based on a single biallelic marker corresponds basically to a dichotomous decision between resistant or susceptible genotypes. We explored thus the outcomes of relaxing the culling levels of genomic prediction (W-BLUP as selection criteria) from 10 to 20%, 30%, 40% and even 50% (Table S1). As a general observation, the more relaxed the culling levels the more accessions were shifted from quadrant (IV) to (III) and from quadrant (I) to (II). For instance, when the culling level was relaxed for selection of the 30% less susceptible BaYMV and BaMMV predictions, most of the suitable accessions (up to 83.8%) according to MAS using 3:690,855,738 have the chance to be selected. Respecting MAS using the most associated marker on 4H for BaMMV, relaxing the culling level to 50% allowed the selection of 35.7% of the total number of accessions that would have been selected by only doing MAS.

## Discussion

### Curating historic data for susceptibility to BaYMV and BaMMV

Past studies have demonstrated the great value of historical data to populate a bio-digital resource center (Gonzalez et al. 2018b; Philipp et al. 2019), to analyze the genetic basis of complex traits with genome-wide association mapping (Milner et al. 2019), and to train genome-wide prediction models (Rutkoski et al. 2015). Thorough data curation is an important prerequisite when using historic records, which has so far been implemented applying standard outlier tests in combination with weather data (Philipp et al. 2018, 2019). We extended the existing strategy and estimated the genomic repeatability of susceptibility to BaYMV and BaMMV for each location and year combination separately, which provided insights into the quality of non-replicated field trials (Fig. 3 and Table 1). The exception were of course very small experiments, such as the screening of only 15 accessions in the year 1985 for BaMMV susceptibility. In this case, the interpretation of genomic repeatability is not straightforward nor free of bias. As a result of the data curation process, a comprehensive and diverse training population was assembled with phenotypic data from six-rowed and two-rowed barley originating from 44 geographic places.

The phenotypic data were used to study the genetic basis of susceptibility to BaYMV and BaMMV applying GWAS. The strong peaks on chromosome 3H and 4H showed the most associated markers detected in a region where previous studies reported the presence of resistance-conferring loci encoding the eukaryotic translation initiation factor *Hv-eIF4E* (*rym4/5*) and *rym13*, respectively (Milner et al. 2019). Moreover, these chromosomes have been a hot spot for bymovirus resistance genes reported in barley. To date, a total of 18 resistance genes have been identified as underlying factors controlling the susceptibility to BaYMV and BaMMV (Jiang et al. 2020). Despite the large population size in our study, the proportion of phenotypic variance explained by single significant markers (up to 8.8%) and by the sum of them (up to 32%) was low, which points to a polygenetic genetic architecture of some major plus several minor loci. This scenario encouraged us to study the potential of genome-wide prediction giving specific weight to major genes.

### Genome-wide prediction of susceptibility to BaYMV and BaMMV

One long-term goal of genebanks is to characterize entire collections for important agronomic and quality traits. This information can then be used in a first step to identify donors of novel beneficial genes and alleles, which are potentially lost in the course of intensive breeding. However, due to the large population sizes and the phenotyping bottleneck, the characterization of entire collections is often limited to smaller subpopulations. We followed the strategy proposed by Yu et al. (2016) and studied the potential of genome-wide prediction to estimate susceptibility to BaYMV and BaMMV. Our findings clearly underlined the suitability of historical data combined across years and locations to achieve high prediction abilities of 0.62 for BaYMV and 0.64 for BaMMV using GBLUP model. Interestingly, GBLUP was outperformed by W-BLUP with minor profits in prediction ability of 0.02 for BaYMV (~ 3.0%) and 0.03 for BaMMV (~ 5.0%) when highly significant markers (first 10 and 20 markers having the lowest *P*-values for associations in GWAS) were modeled as fixed effects in a GS model.

Bernardo (2014) studied through simulations the influence of different trait heritabilities, QTL sizes, and numbers, as well as population sizes, on the differences in prediction accuracy and selection efficiency of genomic prediction by modeling major QTL as fixed effects plus the remaining markers effects as random against modeling all marker effects as random in RR-BLUP. As a rule of thumb, Bernardo (2014) concluded that provided the existence of one to three major QTL, each accounting for at least 10% of the genetic variation underlying moderate to highly heritable traits, modeling them as fixed effects will be in general beneficial. Similar results were reported in a more recent simulation study (Rice and Lipka 2019). Furthermore, this approach was also suitable for disease resistance prediction when both large-effect and small-effect resistance genes are involved (Poland and Rutkosky 2016), while the model GS + de novo GWAS outperformed other models for different traits in rice (Spindel et al. 2016). Particularly, the W-BLUP model used in the current study outperformed the prediction abilities of both MAS and genomic prediction for heading date and plant height in a hybrid wheat population (Zhao et al. 2014). Therefore, we recommend to populate the IPK bio-digital resource center for susceptibility to BaYMV and BaMMV using the W-BLUP model.

Most modern breeding approaches targeting resistance to yellow mosaic virus disease have been based on resistance screenings for the identification of donors and genetic mapping (Perovic et al. 2014; Lupken et al. 2013, Ordon et al. 2004). To the best of our knowledge, there are no reports of genomic predictabilities for these viruses. Particularly, R^2^ values from past linkage mapping studies are usually inflated, which make them difficult to compare with our less biased cross-validated prediction ability results.

Last but not least, accommodating more than 10 and 20 informative markers in W-BLUP for BaYMV and BaMMV prediction decreased the advantage in prediction ability of W-BLUP over GBLUP (Fig. 5). Similar results were observed for a W-BLUP model that accommodated information on major QTL as random factors when more markers were incorporated by relaxing the significance threshold for detected associations underlying different traits in maize (Li et al. 2020). The observed drop in prediction ability was most likely caused by the increased number of false positive associations expected from more liberal thresholds applied during cross-validations. Therefore, we encourage the utilization of peak GWAS associations in an augmented GS model always considering a respective trait-customized assessment of prediction abilities for an optimized number of associations (Fig. 5) prior to its implementation into a selection scheme for genebank material. In this context, other methods to select major QTL for W-BLUP could be further explored. For instance, instead of using the top-n most significant markers as implemented in the present study, a representative marker for each detected QTL region could be selected for their inclusion into W-BLUP in order to evenly cover all genomic regions containing major QTL. Nevertheless, we anticipate that the selection of representative markers will be challenging for QTL defined by large genomic regions with complex LD patterns like, for instance, the one detected on chromosome 3H (Fig. S4).

### Selection of individuals based on W-BLUP and MAS for pre-breeding

MAS (Fig. 4) showed a lower prediction ability than genomic prediction (Fig. 3) for bymovirus resistances. The general superiority of genomic prediction over MAS was already reported in the seminal paper of Meuwissen et al. (2001) and has been documented afterward for several traits in plants, including disease resistances in cereals (Arruda et al. 2016; Mirdita et al. 2015). Nevertheless, since MAS has still a wide application in breeding programs based on backcrossing and pyramiding (Ordon et al. 1999; Werner et al. 2005), resistance (pre-)breeders may be more familiar with this method. Thus, besides prediction ability comparisons we contrasted the outcome of genome-wide and marker-assisted selection in terms of selection decisions when embarking on pre-breeding programs (Fig. 6 and Table S1). As mentioned before, besides being more precise than MAS, one of the main advantages of using genomic predictions as selection criteria lies on their continuous nature. It could be argued that even though shifting culling levels is not possible for MAS, the high variation within the resistant pool (see, for instance, quadrants (III) and (IV) in Fig. 6) intrinsically allows to keep a certain amount of diversity for further subsequent selection steps. Nevertheless, the advantage of doing this based on culling levels applied to genomic predictions is that breeders can informedly control the trade-off between selection intensity and genetic diversity. Breeders can explore more relaxed culling levels to increase the number of accessions selected by genomic prediction that could have been also selected by MAS. For instance, a culling level of 30% for genomic predictions can include most of the candidates following a MAS for BaYMV approach (Table S1). However, the crossing and testing capacities of pre-breeding programs would ultimately determine how far the number of accessions can be increased.

### Comparison between GBLUP and the focused identification of germplasm sources (FIGS) strategy

One alternative approach to select subpopulations that are then phenotyped in intensive trials exploits knowledge on the ecogeographical information of the sites where the accessions were collected such as occurrence of diseases. This approach has been denoted as the focused identification of germplasm sources (FIGS) algorithm (Mackay and Street 2004). The average susceptibility of the accessions examined in our study that originate from East Asia is much lower as compared to accessions from other parts of the world (Fig. 2). This is most likely because of the selection in favor of resistant genotypes in this region, since the first symptoms for the yellow mosaic disease were reported in 1940 in East Asia with a devastating effect on yield in 1970s. This is further substantiated by molecular studies reporting that the donors of 12 resistance genes originated from Japan and China. (For more details about this topic, please read the review Jiang et al. 2020.) Thus, the FIGS approach would also lead from susceptibility to BaYMV and BaMMV to an enrichment in a selected subpopulation. The wide distribution of susceptibilities observed for the East Asian accessions, however, also evidences the limits of the FIGS approach.

As an extension, FIGS was combined with GBLUP (FIGS^+^) by including besides the marker-based additive relationship matrix also a relationship matrix established using bioclimatic variables of the collection site. This FIGS^+^ approach lead to slight but non-statistically significant improvement in a data set of 789 bread wheat landraces that were fingerprinted with 12 K DartSeq SNP markers and scanned for seed morphometric traits (Kehel et al. 2020). It would be interesting to test this approach also with our comprehensive data set, which, however, is hampered by the lack of geo-localization data of the collection site for 93% of the accessions in our study. Thus, the applied approach based on W-BLUP represents, at the moment, the best approach to enrich the information on resistances against barley soilborne mosaic viruses of the bio-digital resource center hosted at the genebank of the Leibniz Institute of Plant Genetics and Crop Plant Research (IPK).

## Supplementary Information

Below is the link to the electronic supplementary material.

Supplementary file1 (DOCX 2657 kb)

## Data Availability

The historical and non-orthogonal data of plant responses to barley yellow mosaic viruses are already published at Milner et al. (2019), and SNP matrices data have been deposited at https://doi.org/10.5447/IPK/2018/9 (Mascher 2018).
