# Supplementary material for: Genomic prediction models trained with historical records enable populating the German ex situ genebank bio-digital resource center of barley (Hordeum sp.) with information on resistances to soilborne barley mosaic viruses
Source: Theor Appl Genet. 2021 Mar 25;134(7):2181–96. doi: 10.1007/s00122-021-03815-0 (PMC8263548; doi:10.1007/s00122-021-03815-0)
Supplement: Supplementary file 1 — Supplementary file1 (DOCX 2657 kb) [file 122_2021_3815_MOESM1_ESM.docx]

**Supplementary material**

**Genomic prediction models trained with historical records enable populating the German ex-situ genebank bio-digital resource center of barley (*Hordeum* sp.) with information on resistances to soilborne barley mosaic viruses**

Maria Y. Gonzalez^1^, Yusheng Zhao^1^, Yong Jiang^1^, Nils Stein^1,2^, Antje Habekuss^3^, Jochen C. Reif^1†^, Albert W. Schulthess^1^

**Author affiliations**

^1^ Department of Breeding Research, Leibniz Institute of Plant Genetics and Crop Plant Research (IPK), D-06466, Gatersleben, Germany

^2^ Department of Genebank, Leibniz Institute of Plant Genetics and Crop Plant Research (IPK), D-06466, Gatersleben, Germany

^†^ To whom correspondence should be addressed. Email: [reif@ipk-gatersleben.de](mailto:reif@ipk-gatersleben.de)

**
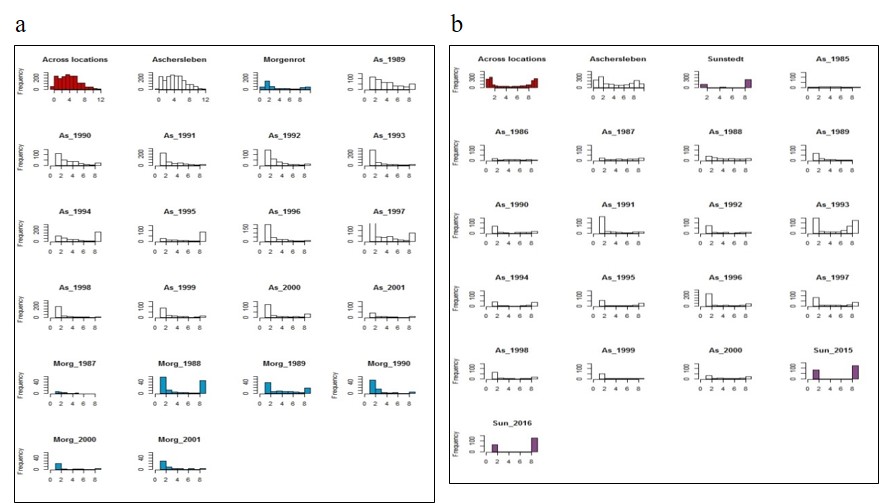
**

**Supplemental Figure S1** Phenotypic distributions of accessions reaction against (**a**) BaYMV and (**b**) BaMMV scored at three locations (As, Aschersleben; Morg, Morgenrot; Sun, Sunstedt) during the 1985-2016 time period. Phenotypic performances are expressed using a 1 (complete absence of symptoms) to 9 (completely susceptible) infection scoring scale and correspond to the Best Linear Unbiased Estimations (BLUEs) computed using three different levels of historical information: (i) across locations and years, (ii) across years within locations, and (iii) within years within locations.


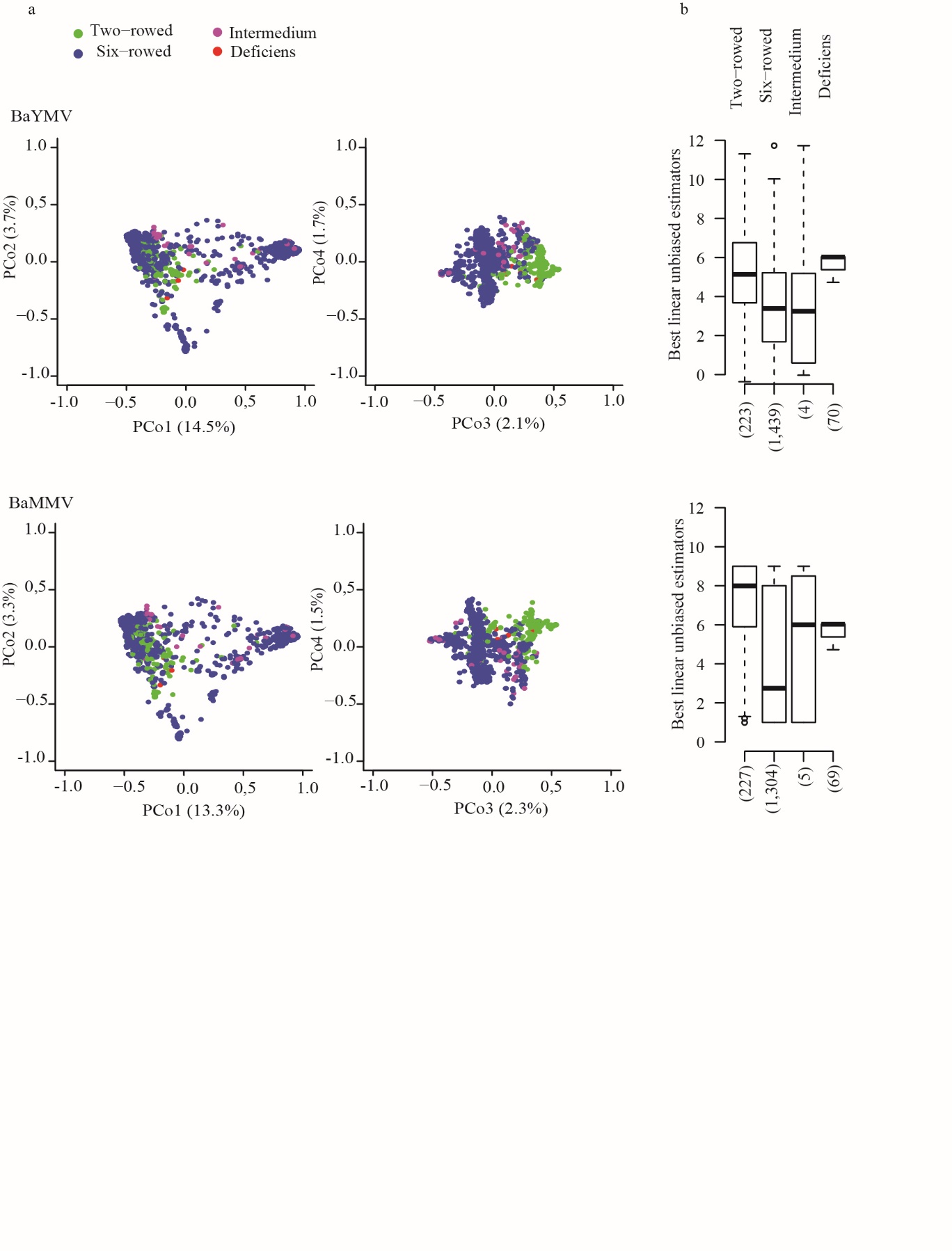

**Supplemental Figure S2** Genetic and phenotypic diversity as a function of row type portrayed by 2,083 accessions assessed for susceptibilities to mosaic viruses BaYMV (upper half) and BaMMV (lower half) at up to 2 locations in 1985 to 2016. (**a**) Biplots considering the first four principal coordinates (PCo) from a principal coordinate analysis performed on pairwise Rogers’ distances matrix among accessions. The different colors represent the row types according to the passport data of accessions. (**b**) Distribution of the Best Linear Unbiased Estimations (BLUEs) of accessions according to their row type. The numbers in brackets refer to the total number of accessions in each geographical origin.


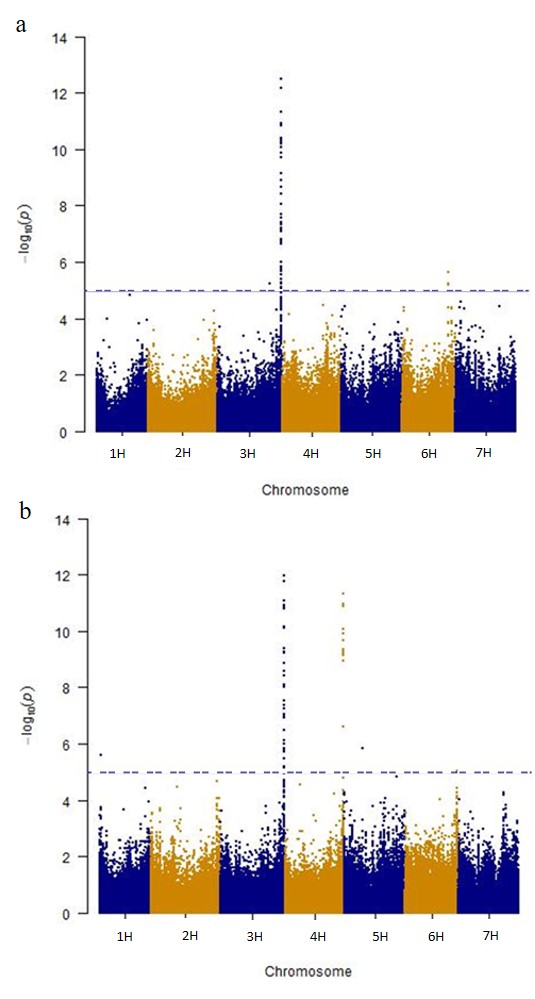


**Supplemental Figure S3** Manhattan plots of the negative logarithm of FDR-corrected $P$-values from genome-wide association scans for susceptibilities to (**a**) BaYMV and (**b**) BaMMV scored for 2,083 accessions at three different locations within the 1985-to-2016 time period. The blue dashed line indicates the significant threshold assuming a nominal α level of 0.05.

**
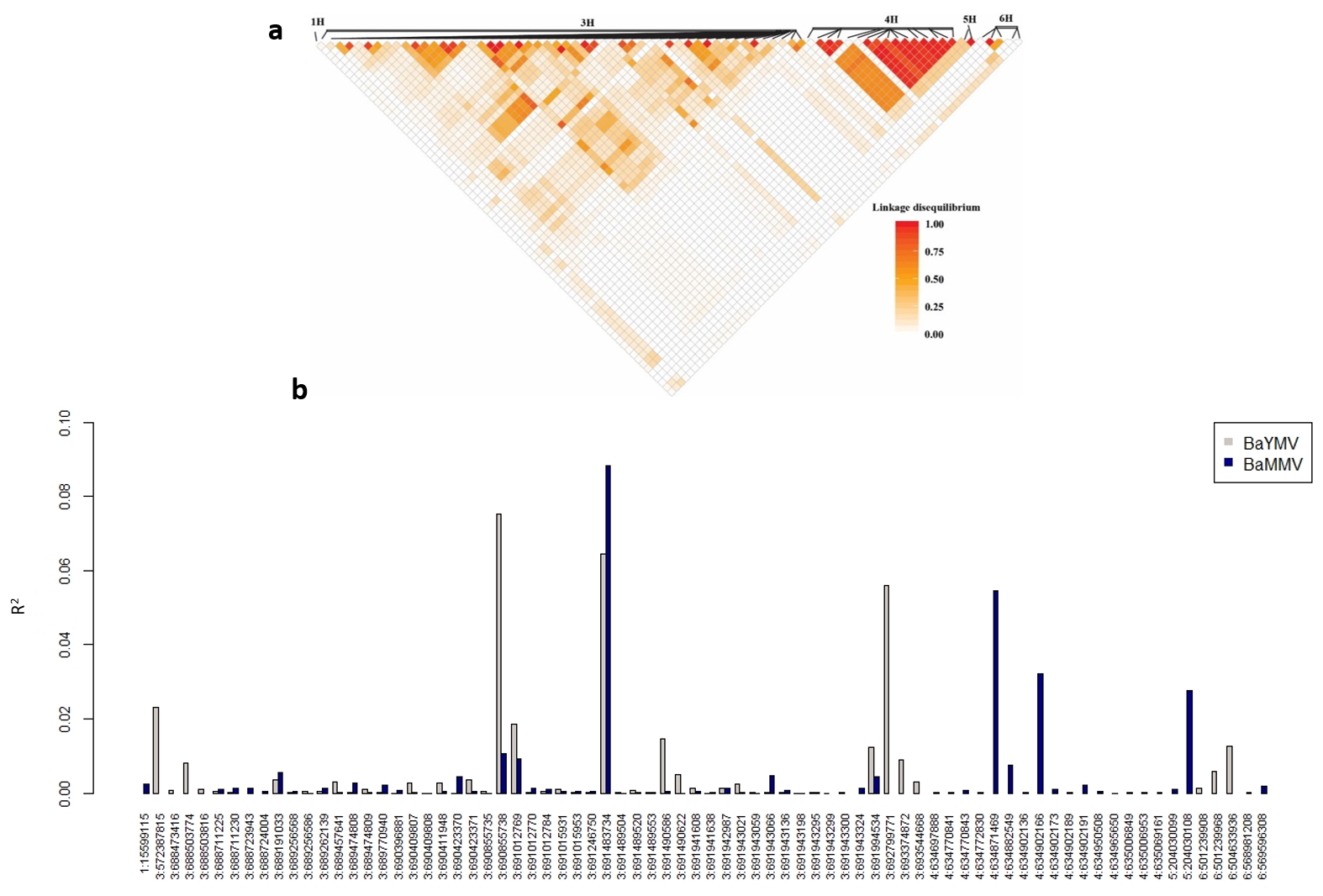
**

**Supplemental Figure S4** (**a**) Linkage disequilibrium ($r^{2}$) and (**b**) adjusted R^2^ for the markers significantly associated to susceptibilities against BaYMV and BaMMV infections using a significant threshold of $P$-value < 0.05.


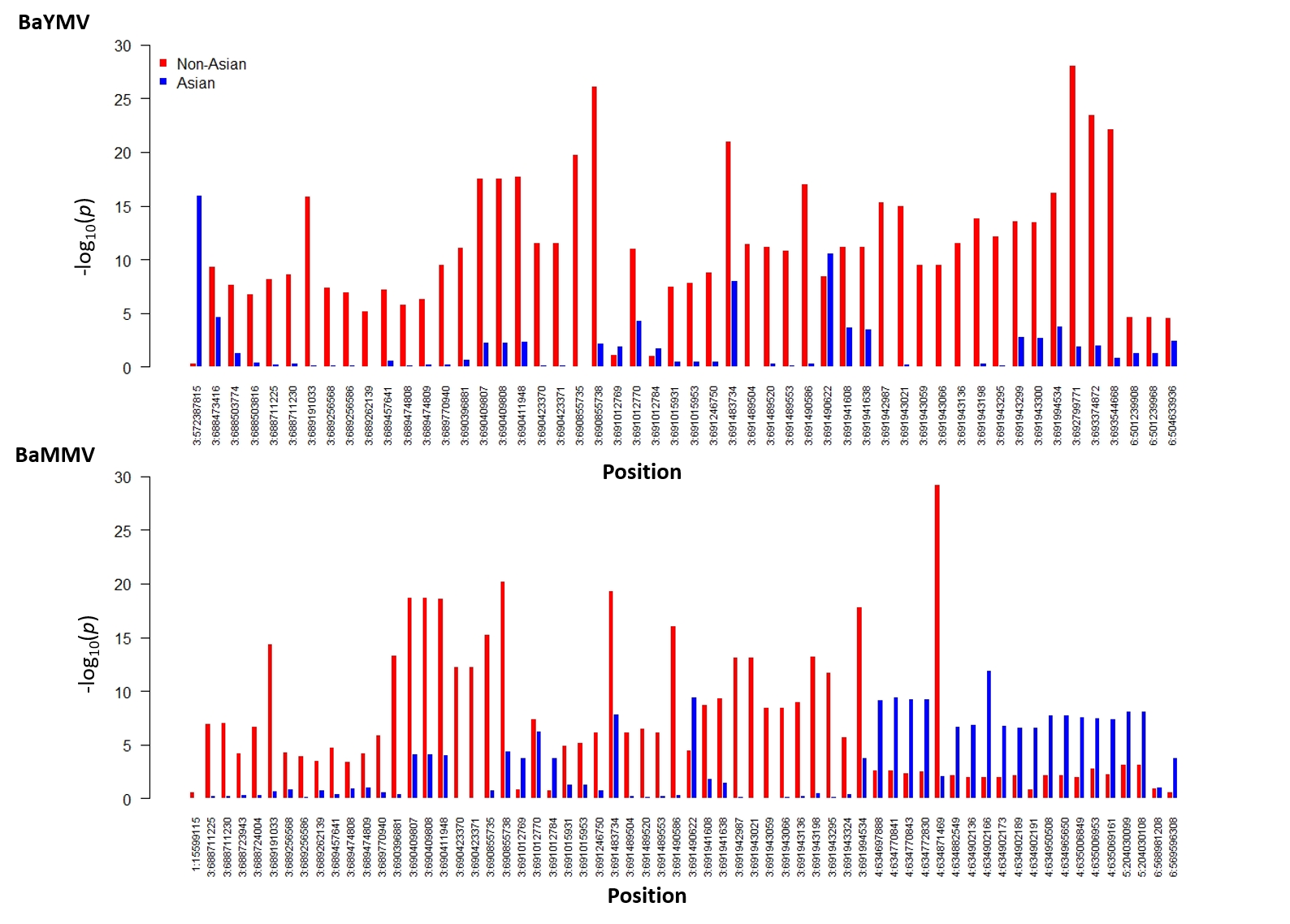


**Supplemental Figure S5.** Negative logarithm of the *P*-value determined in a linear regression for 52 and 64 markers detected as significant in the genome wide association scans for BaYMV (top) and BaMMV (bottom) infections, respectively (see Fig. S3 for more details). The different colors represent the Non-Asian (red) and Asian accessions (blue).


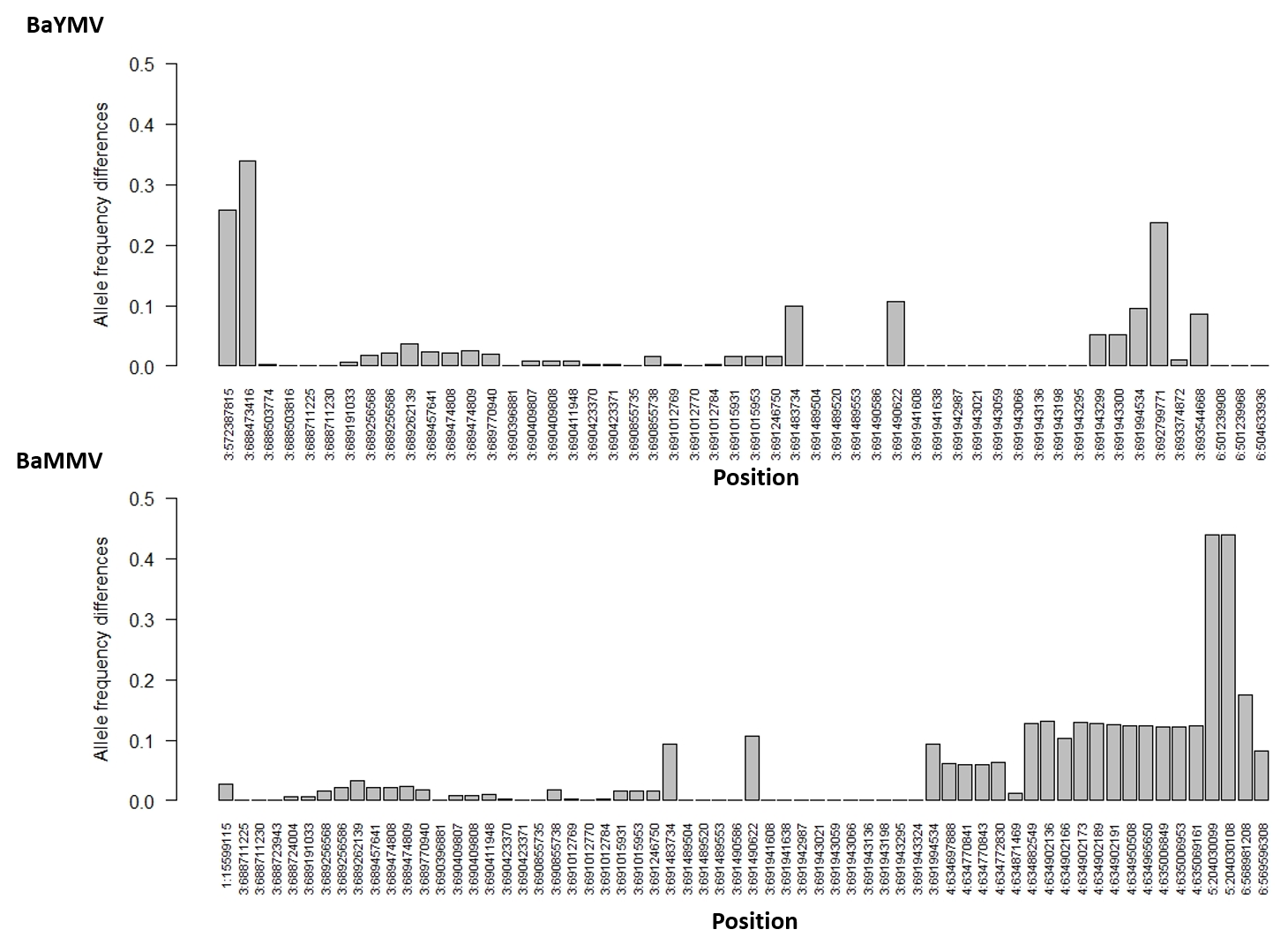


**Supplemental Figure S6** Allele frequency differences between Non-Asian and Asian accessions at 52 and 64 markers detected as significant in the genome wide association scans for BaYMV (top) and BaMMV (bottom) infections, respectively (see Fig. S3 and Fig. S5 for more details).


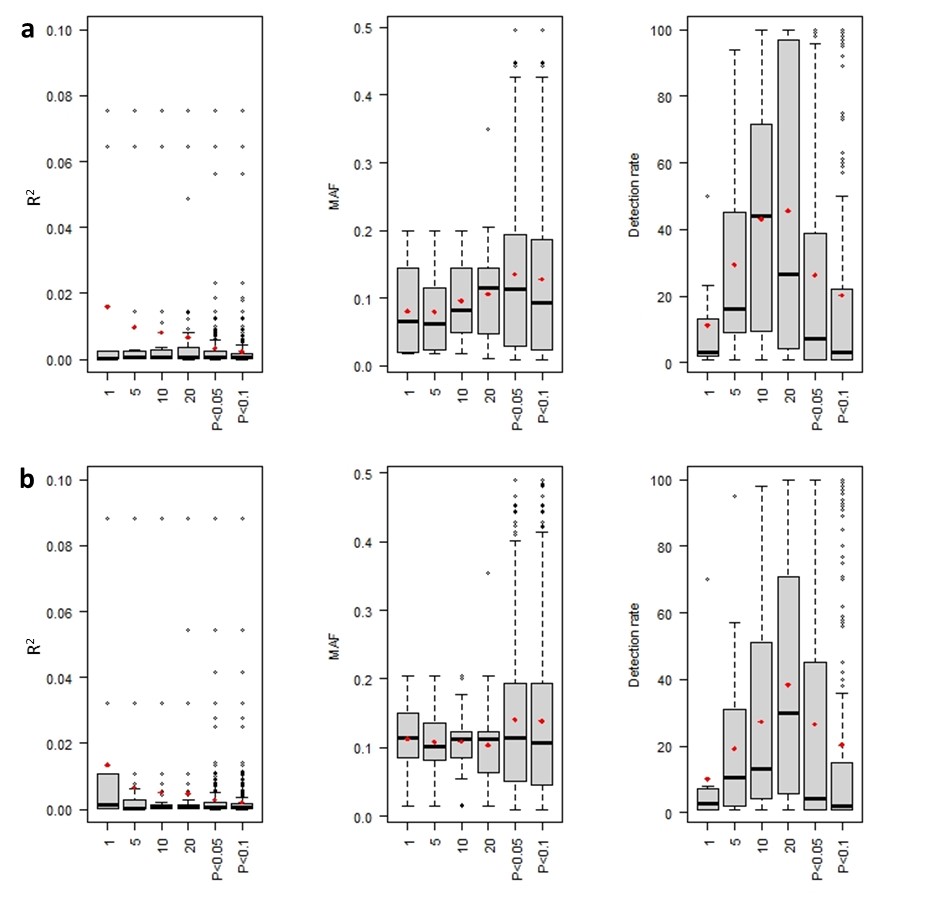


**Supplemental Figure** **S7** Adjusted R^2^, minor allele frequency (MAF), and detection rate of associations found for (**a**) BaYMV, and (**b**) BaMMV susceptibilities according to six significant thresholds: (i) the first, (ii) first 5, (iii) first 10, and (iv) first 20 most significant SNPs, as well as SNPs whose associations were significant at (v) $P$-value < 0.05 and (vi) $P$-value < 0.1. Adjusted R^2^ and MAF were computed in the total dataset while detection rates are based on 100 cross-validations. The red dots represent the mean values.

**
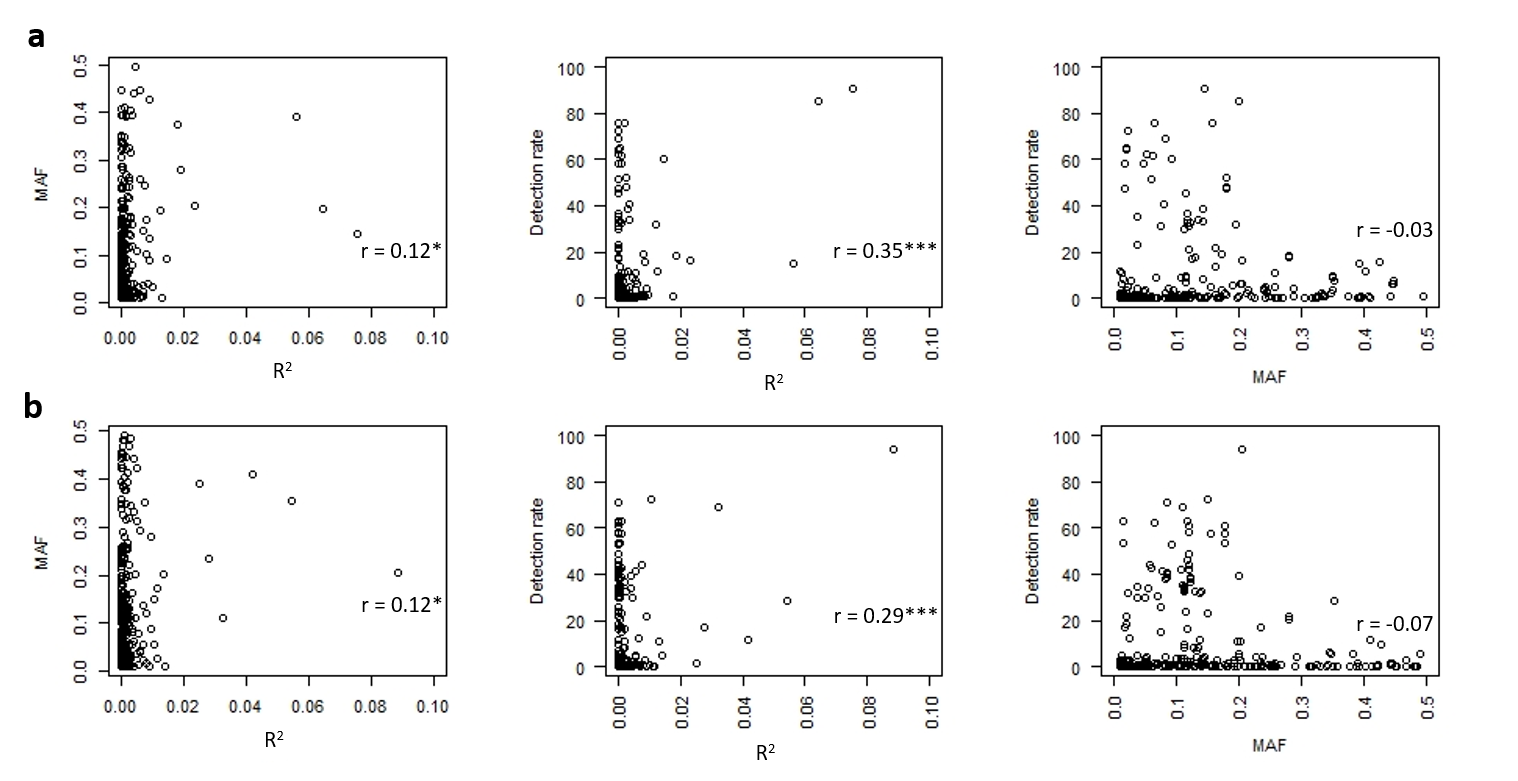
**

**Supplemental Figure S8** Relationship among adjusted R^2^, minor allele frequency (MAF), and detection rate (%) for markers associated at threshold of $P$-value < 0.1 revealed in 100 cross-validations, for (**a**) BaYMV, and (**b**) BaMMV susceptibilities. Detection rate was estimated across scenarios. The Pearson’s correlation coefficient (r) is presented in the lower right corner of each plot. Correlations that significantly differ from zero (p-value < 0.0001) are indicated with ***.

**Supplemental Table S1** Comparison among genome-wide prediction using W-BLUP and marker-assisted selection (MAS) for non-phenotyped winter barley accessions maintained at the IPK genebank. Culling levels separating the 10%, 20%, 30%, 40% and 50% less susceptible genotypes detected by genomic prediction. W-BLUP was based on first 10 SNPs for BaYMV, and first 20 SNPs for BaMMV having the lowest *P*-values for associations during GWAS. MAS stand for the highest associated marker (3:690855738) for BaYMV susceptibility and for the most associated markers at chromosomes 3H (3:691483734) and 4H (4:634871469) for BaMMV susceptibility. Each culling level included the associated phenotypic selection threshold (maximum susceptibility), I-IV quadrants and their respective number of accessions (N), as well as the percentage of accessions (% MAS) selected by genomic prediction and MAS [Q(III)] over the total number of accessions selected by MAS [Q(III) + Q(IV)].

| **Trait** |  | **Culling levels (%)** |  | **Maximum susceptibility** |  | **Quadrants (N)** | | | |  | **% MAS** |
| --- | --- | --- | --- | --- | --- | --- | --- | --- | --- | --- | --- |
|  |  |  |  |  |  | **I** | **II** | **III** | **IV** |  |  |
|  |  |  |  |  |  |  |  |  |  |  |  |
| **BaYMV** |  | 10 |  | 1.66 |  | 1,698 | 111 | 98 | 180 |  | 35.3 |
|  |  | 20 |  | 3.16 |  | 1,574 | 235 | 182 | 96 |  | 65.5 |
|  |  | 30 |  | 3.95 |  | 1,416 | 393 | 233 | 45 |  | 83.8 |
|  |  | 40 |  | 4.5 |  | 1,230 | 579 | 256 | 22 |  | 92.1 |
|  |  | 50 |  | 4.8 |  | 1,029 | 780 | 264 | 14 |  | 95.0 |
|  |  |  |  |  |  |  |  |  |  |  |  |
| **BaMMV**  **3H** |  | 10 |  | 2.05 |  | 1,569 | 239 | 181 | 110 |  | 38.5 |
|  |  | 20 |  | 3.31 |  | 1,409 | 399 | 231 | 60 |  | 62.2 |
|  |  | 30 |  | 4.62 |  | 1,229 | 579 | 261 | 30 |  | 79.4 |
|  |  | 40 |  | 5.54 |  | 1,029 | 779 | 271 | 20 |  | 89.7 |
|  |  | 50 |  | 6.09 |  | 1,569 | 239 | 181 | 110 |  | 93.1 |
|  |  |  |  |  |  |  |  |  |  |  |  |
| **BaMMV**  **4H** |  | 10 |  | 2.05 |  | 1,507 | 365 | 55 | 172 |  | 10.1 |
|  |  | 20 |  | 3.31 |  | 1,323 | 549 | 81 | 146 |  | 24.2 |
|  |  | 30 |  | 4.62 |  | 1,136 | 736 | 104 | 123 |  | 35.7 |
|  |  | 40 |  | 5.54 |  | 945 | 927 | 123 | 104 |  | 45.8 |
|  |  | 50 |  | 6.09 |  | 1,507 | 365 | 55 | 172 |  | 54.2 |
